# Effects of diclofenac on the pharmacokinetics of celastrol in rats and its transport

**DOI:** 10.1080/13880209.2018.1459740

**Published:** 2018-04-13

**Authors:** Zengfu Wang, Dali Chen, Zhongwei Wang

**Affiliations:** a Department of Anesthesiology, Shengli Oilfield Central Hospital, Dongying, P. R. China;; b Department of Laboratory Medicine, Yidu Central Hospital of Weifang, Weifang, P. R. China

**Keywords:** Caco-2 cells, P-gp, LC-MS

## Abstract

**Context:** Diclofenac and celastrol are always used together for the treatment of rheumatoid arthritis; the herb–drug interaction potential between diclofenac and celastrol is still unknown.

**Objective:** This study investigates the effects of diclofenac on the pharmacokinetics of celastrol in rats.

**Materials and methods:** Twelve male Sprague-Dawley rats were divided into two groups and received celastrol (1 mg/kg) or both celastrol (1 mg/kg) and diclofenac (10 mg/kg) by oral gavage, and blood samples were collected via the *oculi chorioideae* vein and determined using the LC-MS method developed in this study. Additionally, the effects of diclofenac on the transport of celastrol were investigated using a Caco-2 cell transwell model.

**Results:** Diclofenac could significantly (*p* < 0.05) decrease the *C*_max_ (from 66.93 ± 10.28 to 41.25 ± 8.06 ng/mL) and AUC_0-t_ (from 765.84 ± 163.61 to 451.33 ± 110.88 μg × h/L) of celastrol in rats. The efflux ratio of celastrol increased significantly (*p* < 0.05) from 3.12 to 4.55 with the treatment of diclofenac.

**Discussion and conclusion:** These results indicated that diclofenac could decrease the system exposure of celastrol in rats when they are co-administered, and these effects might be exerted via decreasing its absorption in intestine.

## Introduction

Celastrol is a major bioactive component extracted from the root bark of the Chinese medicine ‘Thunder of God Vine’ (*Tripterygium wilfordii* Hook F. [Celastraceae]) (Yang et al. 2006). Celastrol has been used for the treatment of autoimmune diseases, such as asthma, chronic inflammation and neurodegenerative disease (Pinna et al. 2004; Astry et al. 2015; Sharma et al. 2015; Yu et al. 2015), and the antitumor activities have also been widely studied in recent years (Dai et al. 2010; Ji et al. 2015; Li et al. 2015; Shrivastava et al. 2015). However, the clinical application of celastrol has been restricted for its narrow therapeutic window and severe toxicity to digestive, reproductive and haematopoietic systems; its toxicity has been verified in both humans and animals (Chai et al. 2011; Wang et al. 2011; Yang et al. 2012; Wang et al. 2013).

Diclofenac, a potent non-steroidal anti-inflammatory drug and one of the most useful pain killers, is used clinically in treatment of rheumatic or non-rheumatic inflammatory diseases (Al-Amin et al. 2013; Guyot et al. 2017). Diclofenac inhibits the conversion of arachidonic acid to prostaglandins which are responsible for the inflammatory process (Orido et al. 2008; Gan 2010; Rowcliffe et al. 2016). P-glycoprotein (*P-gp*) also known as multidrug resistance protein 1 or ATP-binding cassette sub-family B member 1 (ABCB1) is an important protein of the cell membrane that pumps many foreign substances out of cells (Kataoka et al. 2011; Binkhathlan and Lavasanifar 2013). Some research articles have indicated that diclofenac could induce the activity of *P-gp*, and administration of diclofenac and *P-gp* substrate may result in a clinically significant drug–drug interaction (Lagas et al. 2009; Takara et al. 2009; Sanchez-Covarrubias et al. 2014). As Chinese medicines are often co-administered in clinical practice unwittingly or wittingly, and these interactions are significant safety concerns because the pharmacokinetics of drug and/or active constituent of Chinese medicines may be altered by co-administration, severe and perhaps even life-threatening adverse reactions may occur (Niwa et al. 2014; Wang et al. 2015). Diclofenac and celastrol are always used together for the treatment of rheumatoid arthritis in Chinese clinics. As reported by Li et al. (2016), celastrol is a substrate of *P-gp*, and therefore, we suggested that herb–drug interaction between diclofenac and celastrol might occur when they are co-administered. However, to the best of our knowledge, there is little data available about the effects of diclofenac on the pharmacokinetics of celastrol.

This study investigates the effects of diclofenac on the pharmacokinetic profiles of celastrol in rats. The *in vivo* pharmacokinetics of celastrol in rats with or without treatment with diclofenac was determined using a sensitive and reliable LC-MS method. The effects of diclofenac on the transport of celastrol were also investigated in the Caco-2 cell transwell model.

## Materials and methods

### Chemicals and reagents

Celastrol (purity >98%), diclofenac (purity >98%), verapamil (purity >98%) and hydrocortisone (purity >98%) were purchased from the National Institute for the Control of Pharmaceutical and Biological Products (Beijing, China). Acetonitrile and methanol were purchased from Fisher Scientific (Fair Lawn, NJ). Dulbecco’s modified Eagle’s medium (DMEM) and non-essential amino acid (NEAA) solution were purchased from Thermo Scientific Corp. (Logan, UT). Foetal bovine serum (FBS) was obtained from GIBCO BRL (Grand Island, NY). Penicillin G (10,000 U/mL) and streptomycin (10 mg/mL) were purchased from Amresco (Solon, OH). Hanks’ balanced salt solution (HBSS) was purchased from GIBCO (Grand Island, NY). Ultrapure water was prepared with a Milli-Q water purification system (Millipore, Billerica, MA). All other chemicals were of analytical grade or better.

### Animal experiments

Male Sprague-Dawley rats weighing 220–250 g were provided by the experimental animal centre of the Binzhou Medical University (Shandong, China). Rats were bred in a breeding room at 25 °C with 60 ± 5% humidity and a 12 h dark/light cycle. Tap water and normal chow were given *ad libitum*. All of the experimental animals were housed under the above conditions for a 3 day acclimation period and fasted overnight before the experiments. All experimental procedures and protocols were reviewed and approved by the Animal Care and Use Committee of Binzhou Medical University and were in accordance with the National Institutes of Health guidelines regarding the principles of animal care.

### *In vivo* pharmacokinetic study

To evaluate the effects of diclofenac on the pharmacokinetics of celastrol, the rats were divided into two groups of six animals (statistical significance) each as previously reported (Li et al. 2016; Wang et al. 2016). The test group was received diclofenac (at a dose of 10 mg/kg, dissolved directly in normal saline at a concentration of 2 mg/mL) and celastrol (at a dose of 1 mg/kg, dissolved in normal saline containing 0.5% methylcellulose at a concentration of 0.5 mg/mL) by oral gavage, and the control group only received celastrol (1 mg/kg). The dose of diclofenac and celastrol was chosen according to references reported (Yan et al. 2017). Blood samples (250 μL) were collected into heparinized tubes via the *oculi chorioideae* vein at 0.083, 0.33, 0.5, 1, 2, 4, 6, 8, 10, 12, 24 and 36 h after oral administration of drugs. The blood samples were centrifuged at 4000 rpm for 5 min, and the plasma samples obtained were stored at −40 °C until analysis.

### Determination of celastrol using LC-MS

The LC-MS method was performed according to our previously reported Yan et al. (2017). The analysis was performed on an Agilent Series 1100 (Agilent Technologies, Santa Clara, CA) HPLC system and Agilent G1946 single quadrupole mass spectrometer equipped with an electrospray ionization source. An Agilent ChemStation (version B.02.01) for LC/MS was used for data acquisition and processing. The chromatographic analysis of celastrol was performed on a Waters X-Bridge C18 column (3.0 × 100 mm, i.d.; 3.5 μm, MA, USA) at room temperature. The mobile phase was water (containing 0.1% formic acid) and acetonitrile (25:75, v: v) at a flow rate of 0.6 mL/min, and the split ratio was 1:1. The mass spectrometric analysis was performed in positive ion mode with a selected ion monitoring method. The other parameters were as follows: Fragmentor: 100 v; Drying Gas Flowing Rate: 11.0 L/min; Nebulizer Pressure: 45 psi; Drying Gas Temperature: 350 °C; Capillary Voltage: 4000 V. Hydrocortisone was used as an internal standard. The selected ion was *m*/*z* 451.2 for celastrol and *m*/*z* 363.2 for hydrocortisone.

### Plasma sample preparation

Each plasma sample (100 μL) was spiked with 10 μL of the IS (hydrocortisone, 100 ng/mL). The mixture was then extracted with 190 μL of acetonitrile by vortexing for 1 min. After centrifugation at 12,500 rpm for 10 min using Centrifuge (SORVALL, LEGEND MICRO17, Thermo Scientific, Waltham, MA), the supernatants were transferred to new tubes and evaporated to dryness under a stream of nitrogen. The residue was reconstituted in 200 μL of acetonitrile aqueous solution and centrifuged at 15,000 rpm for 15 min. A total of 5 μL of supernatant was injected into the LC-MS system for the quantitative analysis.

### Preparation of standard and quality control samples

A stock solution of celastrol was prepared in acetonitrile at a concentration of 2 mg/mL. The stock solution of IS was prepared in acetonitrile at a concentration of 1 mg/mL. Calibration standard samples for celastrol were prepared in blank rat plasma at concentrations of 0.1, 0.2, 0.5, 1, 2, 5, 10, 20, 50 and 100 ng/mL. The quality control (QC) samples were prepared at low (0.2 ng/mL), medium (5 ng/mL) and high (7.5 ng/mL) concentrations in the same way as the plasma samples for calibration, and QC samples were stored at −40 °C until analysis.

### Method validation

The LC-MS method validation assays were performed according to the United States Food and Drug Administration (FDA) guidelines (FDA 2013).

### Selectivity

The selectivity of the method was investigated by comparing the chromatograms from six different batches of blank rat plasma with the corresponding spiked plasma to exclude interference from endogenous substances and metabolites.

### Linearity of calibration curves and lower limits of quantification (LLOQ)

The calibration curves were obtained using ten concentrations (0.1, 0.2, 0.5, 1, 2, 5, 10, 20, 50 and 100 ng/mL). The linearity of each calibration curve was determined by plotting the peak area ratio (y) of the analyte to IS versus the nominal concentration (*x*) of the analyte with a weighted (1/*x*^2^) least squares linear regression. The lowest plasma level of celastrol on the calibration curves (0.1 ng/mL) was recognized as the LLOQ, and had acceptable accuracy and precision (≤20%).

### Precision and accuracy

The intra-day precision and accuracy of the method were assessed by determining the concentrations of QC samples five times on a single day, and the inter-day precision and accuracy were estimated by determining the concentrations of QC samples over three consecutive days. The relative standard deviation (RSD) and relative error (RE) were used to express the precision and accuracy, respectively.

### Extraction recovery and matrix effects

The extraction recovery was evaluated by comparing the peak areas obtained from extracted spiked samples with those of the post-extracted spiked samples. The matrix effects were evaluated by comparing the peak areas of the post-extracted spiked QC samples with those of corresponding standard solutions. These procedures were repeated for five replicates at three QC concentration levels (0.2, 5 and 75 ng/mL).

### Stability

The short-term stability was evaluated after the exposure of QC samples at room temperature for 12 h. The post-preparative stability was conducted by reanalysing the QC samples after they were stored for 24 h in the autosampler at ambient temperature. To assess the freeze/thaw stability, the plasma samples were examined after three freeze (−40 °C)-thaw (room temperature) cycles. The long-term stability was determined by assessing the plasma samples after storage at −40 °C for 15 days.

### Cell culture

The Caco-2 cell line was obtained from the American Type Culture Collection (Manassas, VA). The Caco-2 cells were cultured in DMEM high glucose medium containing 15% FBS, 1% NEAA and 100 U/mL penicillin and streptomycin. The cells were cultured at 37 °C with 5% CO_2_. For transport studies, the cells at passage 40 were seeded on transwell polycarbonate insert filters (1.12 cm^2^ surface, 0.4 μm pore size, 12 mm diameter; Corning Costar Corporation, MA, USA) in 12-well plates at a density of 1 × 10^5^ cells/cm^2^. Cells were allowed to grow for 21 days. For the first 7 days, the medium was replaced every 2 days, and then daily. The transepithelial electrical resistance (TEER) of the monolayer cells was measured using Millicell ERS-2 (Millipore Corporation, Billerica, MA), and TEER exceeding 400 Ω·cm^2^ was used for the flux experiment. The integrity of the Caco-2 monolayers was confirmed by the paracellular flux of Lucifer yellow, which was <1% per hour. The alkaline phosphatase activity was validated using an Alkaline Phosphatase Assay Kit. The qualified monolayers were used for studies.

### Effects of diclofenac on the transport of celastrol in the Caco-2 cell transwell model

First to investigate the cytotoxicity of celastrol on Caco-2 cell, the Caco-2 cells were seeded in 96-well plates at a density of 4000 cells per well. Twenty-four hour later, the cells were treated with increasing concentrations of celastrol (1–10 μM) and cultured for 2 h. At the end of the treatment, the MTT uptake method for cell viability determination was conducted according to the manufacturer’s protocol (Promega, Madison, WI) and the plate was read at 490 nm.

Before the transport experiments, the cell monolayers were rinsed twice using warm (37 °C) HBSS, then the cells were incubated at 37 °C for 20 min. After preincubation, the cell monolayers were incubated with celastrol in fresh incubation medium added on either the apical (AP) or basolateral (BL) side for the indicated times at 37 °C. The volume of incubation medium on the AP and BL sides was 0.5 mL and 1.5 mL, respectively, and a 100 μL aliquot of the incubation solution was withdrawn at the indicated time points from the receiver compartment and replaced with the same volume of fresh pre-warmed HBSS buffer. The efflux activity of *P-gp* was validated using a typical *P-gp* substrate digoxin (25 μM). The effects of diclofenac or verapamil (*P-gp* inhibitor) on the transport of celastrol were investigated by adding 20 μM diclofenac or verapamil to both sides of the cell monolayers and preincubating the sample at 37 °C for 2 h. In addition, the effects of diclofenac on the efflux of digoxin (25 μM) were also investigated. The permeability of celastrol (2 μM) (which was validated for no obvious toxicity for Caco-2 cells within 2 h) in all of the above conditions for both directions, i.e., from the AP side to the BL side and from the BL side to the AP side, was measured after incubation for 30, 60, 90 and 120 min at 37 °C.

The apparent permeability coefficient (*P*_app_) was calculated using the equation of Artursson and Karlsson:

$$P_{\text{app}}=\left(\text{Δ}Q\text{/Δ}t\right)\text{×}\left[\text{1/}\left(A\text{×}C_{\text{0}}\right)\right],$$

where *P*_app_ is the apparent permeability coefficient (cm/s), Δ*Q*/Δ*t* (μmol/s) is the rate at which the compound appears in the receiver chamber, *C*_0_ (μmol/L) is the initial concentration of the compound in the donor chamber and *A* (cm^2^) represents the surface area of the cell monolayer. Data were collected from three separate experiments, and each was performed in triplicate.

### Statistical analysis

The differences between the mean values were analysed for significance using a one-way analysis of variance. Values of *p* < 0.05 were considered to be statistically significant.

## Results and discussion

### Method validation

**Figure 1.**
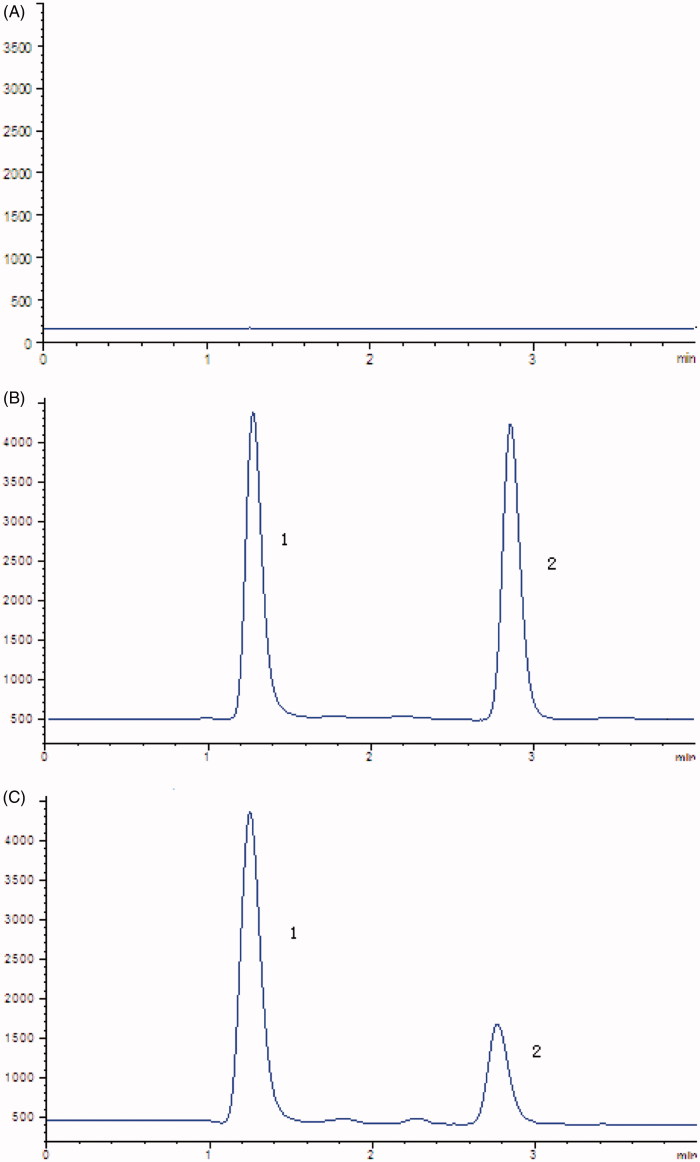
Representative chromatograms of (A) Blank plasma samples; (B) standard solution spiked with celastrol (2) and IS (1); (C) rat plasma samples after oral administration of celastrol.

In the present study, the selectivity was examined using independent plasma samples from six different rats. As shown in Figure 1, no obvious interference was observed in the representative chromatogram of a blank plasma sample at the retention times of the analyte and IS.

Linearity for celastrol was obtained over the concentration range of 0.1–100 ng/mL. The LLOQ of celastrol in rat plasma was 0.1 ng/mL. The precision and accuracy data for the plasma QC samples are presented in Table 1. The intra- and inter-day precision values (RSD) were <10%, and the accuracy (RE) ranged from −10.00% to 8.40%. These data indicated that the accuracy and precision of the method were satisfactory.

**Table 1.** The intra-day and inter-day precision and accuracy of celastrol in plasma samples.

	Intra-day			Inter-day		
Spiked concentration (ng/mL)	Concentration measured (ng/mL)	Precision (%, RSD)	Accuracy (%, RE)	Concentration measured (ng/mL)	Precision (%, RSD)	Accuracy (%, RE)
0.2	0.18	6.28	−10.00	0.21	5.25	5.00
5	5.16	7.62	8.40	4.62	8.37	−7.60
75	79.54	8.31	6.05	71.05	5.10	3.19

Spiked concentration: concentrations of celastrol spiked in plasma samples; Concentration measured: concentration determined by LC-MS; Precision: relative standard deviation of three determinations; Accuracy: relative error of three determinations.

The matrix effects were examined to assess the possibility of ion suppression or enhancement. The matrix effects ranged from 88.62% to 91.27% for celastrol over the three levels of QC samples. The results indicated that no obvious matrix effects were present. The overall mean recoveries of celastrol in plasma at the three different concentration levels were found to be 86.31–3.64% with a RSD <10%, which indicated that the extraction procedure was consistent and reproducible.

The results of the short-term stability, post-preparative stability, freeze and thaw stability and long-term stability are shown in Table 2. It was demonstrated that the stability offered by this method was satisfactory, with the REs and RSD for all samples within the general assay acceptability criteria. These results showed that the samples were sufficiently stable to allow for routine analysis as part of the pharmacokinetic study of celastrol.

**Table 2.** Stability of celastrol in plasma samples (*n* = 3).

Stability conditions	Spiked concentration (ng/mL)	Measured concentration (ng/mL)	Precision (RSD, %)	Accuracy (RE, %)
Short-term	0.2	0.18	5.62	−10.00
(room temperature	5	4.62	7.25	−7.60
for 12 h)	75	68.35	7.01	−8.87
Autosampler (24 h)	0.2	0.21	8.34	5.00
	5	5.37	5.76	7.40
	75	81.98	6.37	9.31
Three freeze/thaw	0.2	0.22	8.46	10.00
cycles (−40 °C)	5	5.43	7.34	8.60
	75	70.26	5.29	−6.32
Long-term (−40 °C	0.2	0.21	6.20	5.00
for 15 days)	5	4.51	8.61	−9.80
	75	69.35	7.29	−7.53

### Effects of diclofenac on the pharmacokinetics of celastrol

**Figure 2.**
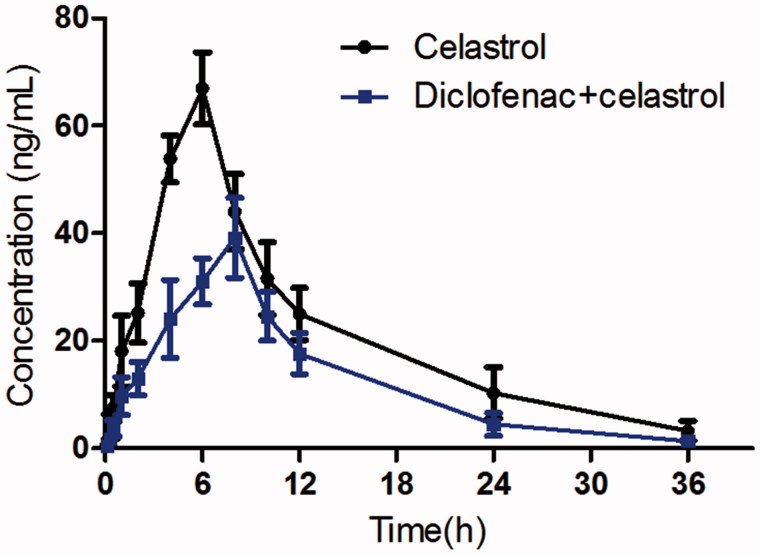
The pharmacokinetic profiles of celastrol in rats after oral administration of celastrol (1 mg/kg) with or without diclofenac (10 mg/kg). Each symbol with a bar represents the mean ± SD of six rats.

The mean plasma concentration–time curves of celastrol with or without diclofenac treatment are presented in Figure 2. The pharmacokinetic parameters of celastrol were calculated using the non-compartmental method with the DAS 3.0 pharmacokinetic software (Chinese Pharmacological Association, Anhui, China). The pharmacokinetic parameters are shown in Table 3.

**Table 3.** Pharmacokinetic parameters of celastrol in rats after oral administration of celastrol (1 mg/kg; *n* = 6, mean ± SD) with or without diclofenac (10 mg/kg).

Parameter	Control	Treatment with diclofenac
*T*_max_ (h)	6.05 ± 1.12	7.68 ± 1.15
*C*_max_ (μg/L)	66.93 ± 10.28	41.25 ± 8.06*
*t*_1/2_ (h)	7.82 ± 1.31	5.93 ± 1.27
AUC_(0-t)_ (μg × h/L)	765.84 ± 163.61	451.33 ± 110.88*
AUC_(0-∞)_ (μg × h/L)	804.60 ± 190.11	462.30 ± 120.71*
*λ* (h^−1^)	0.09 ± 0.02	0.12 ± 0.03
Oral CL (L/h/kg)	1.29 ± 0.15	2.27 ± 0.31*

* *p* < 0.05 indicates significant differences from the control.

When the rats were co-administered with diclofenac, the *C*_max_ of celastrol decreased significantly from 66.93 ± 10.28 to 41.25 ± 8.06 ng/mL (*p* = 0.002), the AUC_0-t_ also decreased significantly from 765.84 ± 163.61 to 451.33 ± 110.88 μg × h/L (*p* = 0.001). The *T*_max_ value of celastrol increased significantly from 6.05 ± 1.12 to 7.82 ± 1.15 h (*p* = 0.002). The oral clearance of celastrol increased from 1.29 ± 0.15 to 2.27 ± 0.31 L/h/kg, and the difference was significant (*p* = 0.001). These results indicated that diclofenac could decrease the system exposure of celastrol in rats when they are co-administered.

As we know, diclofenac and celastrol are always used together for the treatment of rheumatoid arthritis in China clinics, and we infer that diclofenac could increase its clinical safety through decreasing the system exposure of celastrol when they are co-administered.

Previous studies have also reported that diclofenac could significantly decrease the plasma concentration of drugs that co-administered with diclofenac, and it might exert these effects through increasing its intestinal absorption via inducing the activity of *P-gp* (Chew et al. 2017; Yu et al. 2017). Celastrol is a substrate of *P-gp* (Li et al. 2016; Yan et al. 2017), and therefore, we infer that diclofenac might also decrease the system exposure of celastrol through inducing the activity of *P-gp* as *P*-*gp* could hinder its absorption in intestine.

### Effects of diclofenac on the absorption of celastrol across a Caco-2 cell transwell model

**Figure 3.**
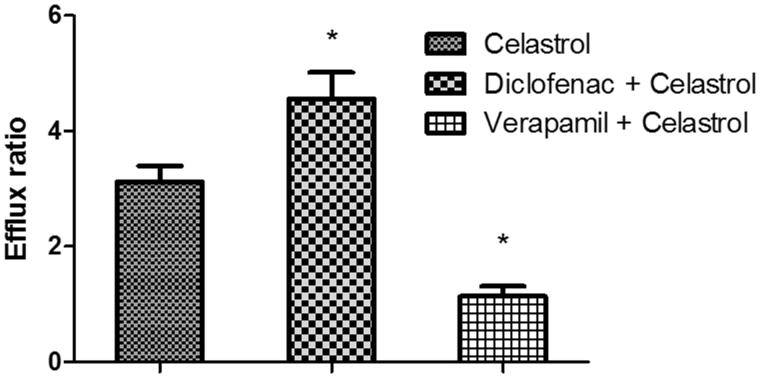
The effects of diclofenac or verapamil on the efflux ratio of celastrol in a Caco-2 cell transwell model. Each symbol with a bar represents the mean ± SD of three determinations. *p < 0.05 indicates significant differences from the control group.

MTT results showed no significant cytotoxicity with celastrol at 2 μM, for 98% cells remained viable. To investigate the effects of *P-gp* on the transport of celastrol, the Caco-2 cell transwell model was used. First, a typical *P-gp* substrate (digoxin) was used to validate the efflux activity of *P-gp*, and the results indicated that the efflux ratio of digoxin was 12.56 (*P*_appAB_: 2.12 ± 0.31 × 10^−7 ^cm/s; *P*_appBA_: 2.66 ± 0.28 × 10^−6 ^cm/s), which was abrogated in the presence of a typical *P-gp* inhibitor verapamil (efflux ratio of 1.49). Then the transport of 2 μM of celastrol across Caco-2 cell monolayers was investigated. As shown in Figure 3, the *P*_appAB_ (apparent permeability coefficient from AP side to BL side) and *P*_appBA_ (apparent permeability coefficient from BL side to AP side) were 5.26 ± 0.73 × 10^−7 ^cm/s and 1.64 ± 0.29 × 10^−6 ^cm/s, respectively. The *P*_appBA_ was much higher than the *P*_appAB_, and the efflux ratio of was 3.12, which indicated that efflux transporters might be involved in the transport of celastrol. The *P*_appAB_ of celastrol was much higher than digoxin, and the efflux ratio of celastrol was lower than digoxin. These results indicated that the absorption of celastrol was much better than digoxin. Then, the transport studies were performed in the presence of diclofenac and verapamil to determine its effects on the transport of celastrol. In the presence of 20 μM of diclofenac, the *P*_app_ values from the AP side to the BL side decreased (4.13 ± 0.55 × 10^−7 ^cm/s), whereas those from the BL side to the AP side increased (1.88 ± 0.31 × 10^−6 ^cm/s). The efflux ratio of celastrol increased significantly from 3.12 to 4.55 (*p* < 0.05). However, in the presence of verapamil (20 μM), a typical *P-gp* inhibitor, the *P*_app_ values from the AP side to the BL side increased (1.21 ± 0.13 × 10^−6 ^cm/s), whereas those from the BL side to the AP side decreased (1.06 ± 0.18 × 10^−6 ^cm/s). The efflux ratio of celastrol decreased significantly from 3.12 to 1.14 (*p* < 0.05). These result indicated that the *P-gp* was involved in the transport of celastrol in the Caco-2 cell transwell model, and diclofenac could enhance the efflux of celastrol in a Caco-2 cell monolayer model via inducing the activity of *P-gp*.

The Caco-2 cell transwell experiments indicated that *P-gp* hindered the absorption of celastrol in intestine, and diclofenac could decrease its absorption through inducing the activity of *P-gp*. Therefore, when the rats were pre-treated with diclofenac, the absorption of celastrol was significantly decreased. These results indicated that the herb–drug interaction between diclofenac and celastrol might occur when they were co-administered.

## Conclusions

Diclofenac could decrease the system exposure of celastrol in rats when they are co-administered, and these effects might be exerted via inducing the activity of *P-gp*. These results also suggest that diclofenac could increase its clinical safety through decreasing the system exposure of celastrol when they are co-administered.
